# Impact of Plasma Rich in Growth Factors (PRGF) Eye Drops on Ocular Redness and Symptomatology in Patients with Dry Eye Disease

**DOI:** 10.3390/medicina59050928

**Published:** 2023-05-11

**Authors:** Javier Lozano-Sanroma, Alberto Barros, Ignacio Alcalde, Ronald M. Sánchez-Ávila, Juan Queiruga-Piñeiro, Luis Fernández-Vega Cueto, Jesús Merayo-Lloves

**Affiliations:** 1 Instituto Oftalmológico Fernández-Vega, 33012 Oviedo, Spain; javilo@fernandez-vega.com (J.L.-S.); alberto.barros@fernandez-vega.com (A.B.); juan.queiruga@fernandez-vega.com (J.Q.-P.); merayo@fio.as (J.M.-L.); 2 Instituto Universitario Fernández-Vega, Fundación de Investigación Oftalmológica, Universidad de Oviedo, 33012 Oviedo, Spain; 3 Instituto de Investigación Sanitaria del Principado de Asturias (ISPA), 33011 Oviedo, Spain; 4 Regenerative Medicine Laboratory, Biotechnology Institute (BTI), 01007 Vitoria, Spain; rsanchez@bti-health.com; 5 Department of Surgery and Medical-Surgical Specialties, Universidad de Oviedo, 33006 Oviedo, Spain

**Keywords:** plasma rich in growth factors, PRGF, ocular inflammation, DED, ocular redness

## Abstract

*Background and Objectives*: Dry eye disease (DED) is a common and very symptomatic pathology that affects normal daily activity. The aim of the study was to evaluate the efficacy of plasma rich in growth factors (PRGF) added to one routine treatment protocol for DED (artificial tears substitutes, lid hygiene, and anti-inflammatory therapy). *Materials and Methods*: Patients were divided into two groups of treatment: standard treatment group (*n* = 43 eyes) and PRGF group (*n* = 59). Patients’ symptomatology (inferred from OSDI and SANDE questionnaires), ocular inflammation, tear stability, and ocular surface damage were analyzed at baseline and after 3 months of treatment. *Results*: OSDI test scores were significantly lower in both groups (*p* < 0.001). SANDE frequency test scores also improved statistically, with differences between groups (*p* = 0.0089 SANDE frequency and *p* < 0.0119 SANDE severity). There was a greater reduction in ocular redness (ocular inflammation) in the PRGF group (*p* < 0.0001) and fluorescein tear break-up time was significantly improved in the PRGF group (*p* = 0.0006). No significant changes were found in terms of ocular surface damage. No adverse events were obtained in either group. *Conclusions*: The addition of PRGF to the standard treatment of DED, according to the results obtained, proved to be safe and produced an improvement in ocular symptomatology and signs of inflammation, particularly in moderate and severe cases, when compared to standard treatment.

## 1. Introduction

Dry Eye Disease (DED) is a disorder that encompasses multiple alterations in the normal functioning parameters of the eye [1] with a high prevalence, estimated to be between 5% and 50% of adults [2], and is characterized by a loss of tear film homeostasis accompanied by ocular symptoms in which tear film instability, hyperosmolarity, ocular surface inflammation, damage, and neurosensory abnormalities play an etiologic role [3].

DED may be caused by excessive tear evaporation, a defect in tear production, or a combination of both. This leads to hyperosmolarity, inflammation, and tissue damage [4]. Therefore, tear film instability, epithelial damage, and ocular surface inflammation will always be present to some extent in DED [5]. These changes, in addition to changes in the palpebral margin and damage to the sensory innervation, are also known as the vicious circle of ocular surface disease [6].

The Management and Treatment Report [7] of the Tear Film and Ocular Surface Society at the Dry Eye Workshop (TFOS DEWS II) suggested that the treatment of tear insufficiency with artificial tear substitutes, which may include aqueous supplementation, tear viscosity optimizers (hyaluronic acid, carboxymethylcellulose, and their combinations: hydroxypropylmethylcellulose), osmotic agents, lipid supplementation, and biological tear substitutes within this group was autologous platelet preparations [8,9,10,11,12].

Biological substitutes include autologous serum (AS) and platelet derivatives [13]. The use of AS for DED appeared in the 1970s [14]. Compared to physiological tears, AS has a similar pH and osmolarity, but has a higher concentration of lysozyme, vitamin A, TGF-β, and fibronectin, which would justify the contribution of AS in maintaining a healthy ocular surface. AS shows a lower level of IgA, EGF, and vitamin C than normal tears [15,16].

Another recently used hematic derivative is plasma rich in growth factors (PRGF). There are several ways of preparation, up to 30, according to the literature [13]. One of those is PRGF Endoret^®^, which is a methodological and standardized preparation [17,18]. PRGF has the advantage over AS of eliminating pro-inflammatory factors released by leukocytes, including IL-6, IL-1B, and TNF-α, which are related to the inflammatory processes of some ocular diseases [19,20]. Thus, PRGF is a plasma serum rich in growth factors enriched in platelets, up to twice as much as AS. Once activated, it could secrete PDGF, TGF-β, VEGF, and IGF-I. As its origin is autologous, the possible immunological reaction is minimized [21]. Another feature of PRGF is its versatility; in addition to being able to formulate PRGF in eye drops, injectable fibrin scaffold and a biodegradable fibrin membrane can also be synthesized [22]. The use of PRGF in DED has already been examined previously in two studies [23,24].

According to the DEWS II, the increasing availability of a variety of topical biologics opens up many new opportunities to investigate the role of these new topical agents in improving a wide variety of symptoms and signs in patients with DED.

The purpose of this study is to evaluate the effect of adding PRGF to the standard DED treatment protocol in ocular surface symptomatology and redness in patients diagnosed with DED.

## 2. Materials and Methods

This observational, longitudinal study was performed at the Ocular Surface Unit of the Instituto Oftalmológico Fernández-Vega (IOFV) in Oviedo (Principality of Asturias, Spain) between May and October 2022. Informed consent was obtained from all patients included in the study. The study was approved by the Research Ethics Committee of the Principality of Asturias, with CEImPA number 2022.168. The study adhered to the tenets of the Declaration of Helsinki.

All patients included in the study were diagnosed with DED. The criteria for selecting cases were that they were ≥18 years old, had an OSDI ≥ 13, and met one of two requirements: non-invasive tear break-up time (FBUT) < 10 s or ocular surface staining 1 on the Oxford fluorescein staining scale [25].

Ocular or systemic pathology that could lead to vision loss such as advanced glaucoma, macular degeneration, retinal detachment, diabetes, among others, was excluded. Those that had discontinued the prescribed treatment were also excluded.

### 2.1. Treatments

All patients discontinued their previous treatments for DED 1 week before starting the new treatment. Patients in the standard treatment group (*n* = 22 patients) were treated with a palpebral hygiene protocol, including eyelid cleansing with Lephanet brand wipes (Laboratoires Théa, Clermont-Ferrand, France) once a day to treat eyelid abnormalities, and this was considered as an inclusion criterion for this study. Ocular hydration supplementation with artificial tears containing hyaluronic acid (Thealoz DUO, Laboratoires Théa) 3 times a day for lacrimal insufficiency, for 3 months, and a top-down regimen (4 times a day for 1 week, 3 times a day for 1 week, twice daily for 1 week, and once a day for 1 week) with fluorometholone 1 mg/mL (FML, Allergan Pharmaceuticals Ireland, Westport, Ireland), for ocular inflammation.

In addition to the standard treatment described above, a group of 30 patients was additionally treated with PRGF 4 times daily for 3 months.

For analytical management, the patients receiving PRGF were referred to as the PRGF group and the others as the standard group.

#### PRGF Preparation

The patients’ blood was collected in 9 mL tubes after an informed consent form was signed. Blood samples from the PRGF were centrifuged at 580× *g* for 8 min at room temperature in an Endoret System centrifuge (BTI Biotechnology Institute, S.L., Miñano, Álava, Spain); the whole plasma column above the buffy coat was collected using an Endoret ophthalmology kit (BTI Biotechnology Institute, S.L., Miñano, Álava, Spain), avoiding the layer containing leukocytes. Platelet and leukocyte counts were performed using a hematology analyzer (Micros 60, Horiba ABX, Montpellier, France). Plasma preparations were incubated with an Endoret activator (BTI Biotechnology Institute, S.L., Miñano, Alava, Spain) at 37 °C for 1 h, and PRGF supernatants were filtered, aliquoted, and stored at −80 °C until use. All procedures were performed under highly sterile conditions in a laminar flow hood. Prior to treatment, patients were instructed to store the PRGF eye drop dispensers at –20 °C for a maximum of 3 months, and each dispenser was used for 3 consecutive days [17,26,27].

### 2.2. Measures Analyzed

#### 2.2.1. Ocular Surface Symptom Assessment

The Ocular Surface Disease Index (OSDI) [28], a 12 item questionnaire that assesses discomfort, visual impairment, and environmental triggers, was used to evaluate the symptoms reported by the patients. The OSDI formula was used for analysis, which is = (sum of scores ×25)/(number of responses).

The Symptoms Analysis in Dry Eye (SANDE) questionnaires [29] were also used to assess the severity and frequency of the symptoms. Patients were asked to rate the severity and frequency of symptoms on a scale of 0 to 100 mm.

To analyze the symptomatology and inflammatory response to treatment, patients were further divided into groups according to their initial OSDI score: Mild (OSDI = 13 to 22), Moderate (OSDI = 23 to 32), and Severe (OSDI > 33) [30].

#### 2.2.2. Visual Acuity

Objective examination was performed with retinoscopy followed by subjective correction. Best corrected visual acuity (BCVA) was recorded on a decimal scale and then converted to log MAR for statistical analysis.

#### 2.2.3. Frequency of Blinks

The LipiView^®^ interferometer (Johnson & Johnson, Stamford, CT, USA) was used to measure blink intervals.

#### 2.2.4. Ocular Redness

To analyze the ocular surface of the inflammation, the degree of ocular redness was measured with photographic capture and subsequent automatic computer analysis using the so-called Oculus Index, with a Keratograph 5M device (Oculus^®^, Wetzlar, Germany). This system automatically generates a bulbar redness score, between 0.0 and 4.0, based on the area percentage ratio between the vessels and the rest of the analyzed area [31,32,33,34].

#### 2.2.5. Fluorescein Staining

To analyze the damage of the ocular surface, a drop of fluorescein (minims fluorescein sodium 20 mg/mL eye drop solution, Laboratoire Chauvin Z.I. Ripotier 07200 Au-benas-France) was instilled and applied to the far temporal part of the eye, while looking upwards to avoid damaging the conjunctival or corneal tissues. The Oxford scale was used.

#### 2.2.6. Stability of the Tear Film

This was analyzed with the FBUT, which is the time, in seconds, that it takes to observe the break-up of the fluorescein-stained tear pattern [1].

#### 2.2.7. Tear Volume

Tear film volume was determined by Schirmer’s test under topical anesthesia. The tip of the strip was inserted into the inferior temporal canthus with the lower eyelid slightly pulled temporally while looking upwards to avoid damage to the conjunctiva, cornea, or lid margin. The strip was left in place for 5 min and the number of millimeters it had been impregnated was measured [35,36].

The same tests were repeated at 3 months.

### 2.3. Statistical Analysis

The data were analyzed using the GraphPad Prism 8 for MacOS (GraphPad Software, San Diego, CA, USA) software and the Effect Size Calculator (Centre for Evaluation & Monitoring, Cambridge University Press, University of Cambridge, Cambridge, UK) [37]. Descriptive statistics were performed on demographic variables.

The normality of the sample was tested using the Shapiro–Wilks and Kolmogorov–Smirnov tests.

To compare quantitative variables before and after treatment, we used Student’s *t*-test for paired samples when normality was met and the Wilcoxon test when normality was not met.

For categorical variables, the Wilcoxon test was used for paired samples and the Mann–Whitney U test was used for independent samples.

To compare groups, the Student’s *t*-test was used for independent samples if they followed a normal distribution and the Mann–Whitney U test if they did not.

*p* < 0.05 was considered statistically significant.

Effect size was calculated using Hedges’ g [38].

## 3. Results

A total of 102 eyes were analyzed; 59 eyes were in the PRGF group (3 males and 27 females) and 43 eyes were in the Standard group (9 males and 13 females). The demographic data and comparison of the variables considered as inclusion criteria are shown in Table 1.

None of the patients had previously received PRGF or platelet-based treatment. All patients had been treated with artificial tears and/or eyelid cleaning, and 20% of the patients in the PRGF group had received short-term step 2 treatment [7] with topical corticosteroids. One patient in the PRGF group had also received cyclosporine and punctal plugs (see Table 2).

The variables analyzed are shown in Table 3. We observed that the OSDI test scores and ocular redness were higher in the PRGF group at baseline, but no statistically significant differences were found (*p* = 0.151 and *p* = 0.773, respectively). No differences were found in corneal staining or FBUT value (*p* = 0.449 and *p* = 0.106, respectively). The total number of blinks was also higher in the PRGF group at baseline, but in this case the difference was statistically significant (*p* = 0.028).

No side effects or intolerances were reported in either group.

**Figure 1:**
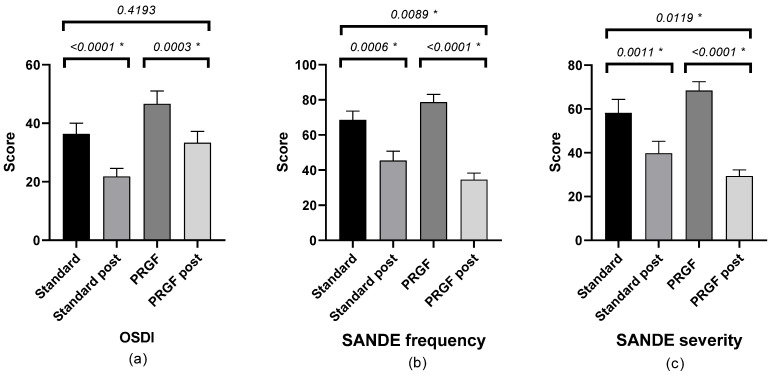
Mean and SEM of the questionnaires (**a**) OSDI, (**b**) SANDE frequency, and (**c**) SANDE severity, initial and after treatment (post) in the standard and PRGF groups. * Statistically significant difference (*p* < 0.05).

There was a statistically significant improvement in symptomatology after treatment in both groups, with a difference between groups according to the SANDE scale (see Figure 1).

**Figure A1:**
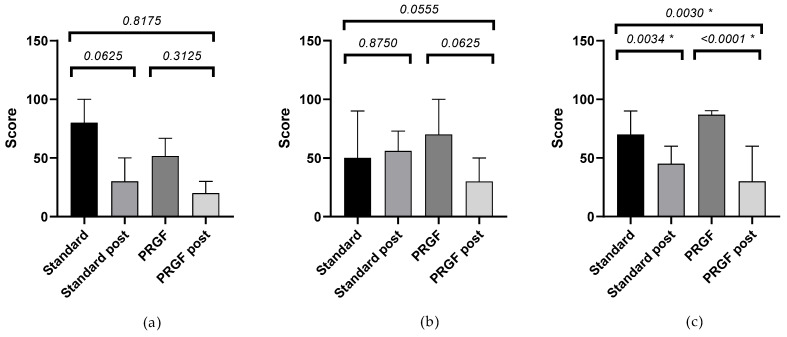
Mean and SEM of the SANDE frequency questionnaire when baseline OSDI was mild (**a**), moderate (**b**), and severe (**c**), initially and after treatment (post) in the standard and PRGF groups. * Statistically significant difference (*p* < 0.05).

**Figure A2:**
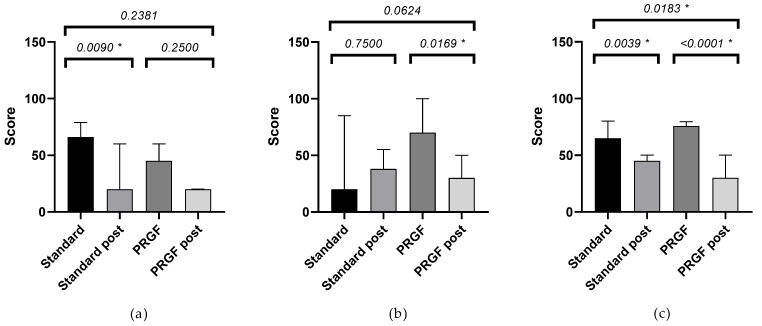
Mean and SEM of the SANDE severity questionnaire when baseline OSDI was mild (**a**), moderate (**b**), and severe (**c**), initially and after treatment (post) in the standard and PRGF groups. * Statistically significant difference (*p* < 0.05).

Table A1 shows the outcome of the SANDE questionnaires. Frequency (Figure A1) and severity (Figure A2) improved (*p* < 0.05) when the initial OSDI questionnaire score was severe, with statistically significant differences between groups.

**Figure 2:**
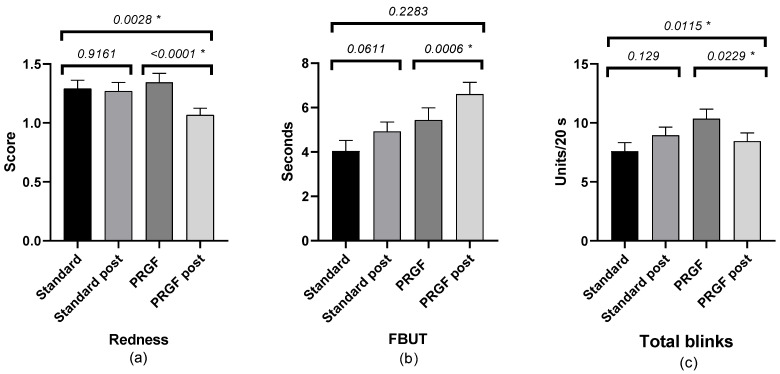
Mean and SEM (Standard Error of Mean) of ocular redness: (**a**) FBUT, (**b**) number of total blinks, and (**c**) initial and after treatment in the standard and PRGF groups. * Statistically significant difference (*p* < 0.05).

**Figure 3:**
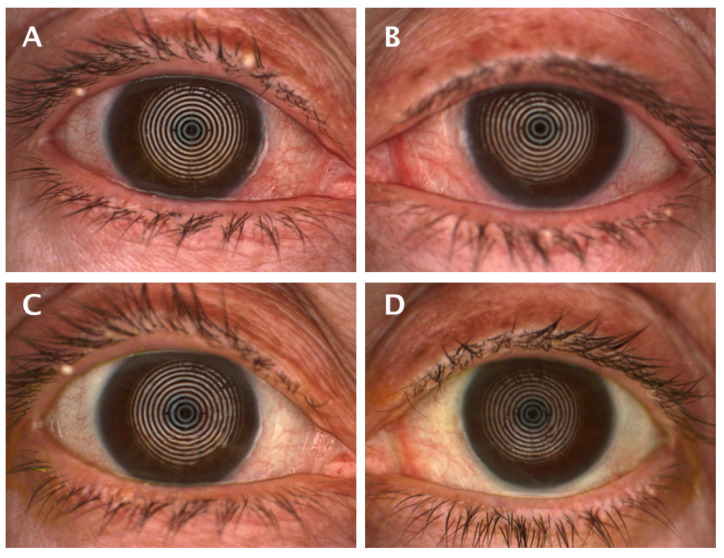
Appearance of ocular redness at the baseline visit with a score of 2.4 (**A**) and 2.1 (**B**), and after treatment 1.4 (**C**) and 1.5 (**D**) in the same patient of the PRGF group.

**Figure A3:**
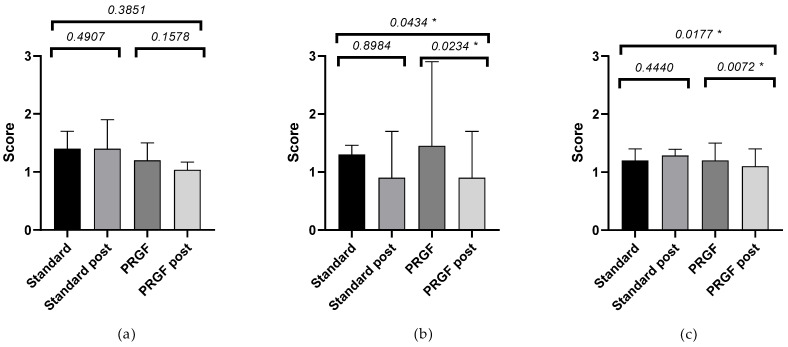
Mean and SEM of ocular redness when baseline OSDI was mild (**a**), moderate (**b**), and severe (**c**), initially and after treatment (post) in the standard and PRGF groups. * Statistically significant difference (*p* < 0.05).

The FBUT value was increased in the PRGF group, with no statistical differences compared to the standard group. There was a statistically significant improvement in the PRGF group in terms of total number of blinks and ocular redness (Figure 2). When ocular redness was studied according to the OSDI score at baseline, a significant improvement was found in moderate and severe cases (see Table A2 and Figure 3 and Figure A3).

**Figure 4:**
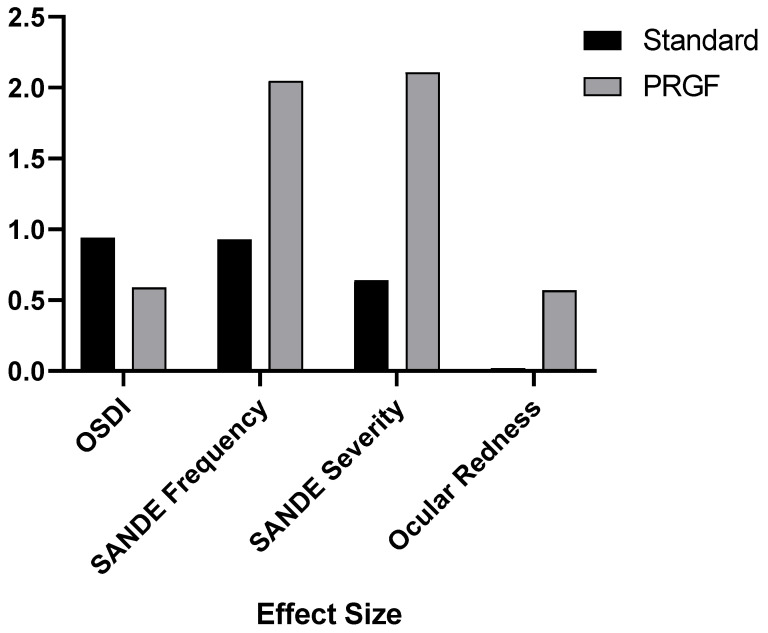
Effect size after treatment with respect to the baseline visit for the variables OSDI, SANDE Frequency and Severity, and ocular redness.

Figure 4 shows how the addition of PRGF affected the variables analyzed by the effect size. It was observed that the effect size was significantly larger for the SANDE symptomatology and ocular redness questionnaires.

## 4. Discussion

The use of PRGF as a treatment for various ocular disorders, such as evaporative dry eye and epithelial disorders, among others, has been described [11,17,23,24,26,27,39,40,41,42,43]. In our study, a wide range of variables were analyzed, both subjective and objective, comparing a group that had received the standard treatment applied in the IOFV and another to which PRGF had been added. For this purpose, inferential statistics were performed, and the effect size of the variables analyzed was studied, as well as the evolution of the frequency and severity of the symptoms reported by the patients and ocular inflammation as a function of the severity of the initial symptomatology, as measured by the OSDI questionnaire.

All patients were treated with supplemental hydration and eyelid hygiene as they had low FBUT and Schirmer values and some degree of Meibomian gland dysfunction, as expected in patients diagnosed with DED. Patients who had more symptoms or had failed at least step 2 [7] treatments were additionally treated with PRGF, according to the ophthalmologist’s criteria.

By analyzing the results, we observed that both groups of patients improved their symptomatology as measured by the OSDI test after receiving the prescribed treatment. This indicates that the combined treatment of palpebral hygiene, hydration and corticosteroids led to an improvement in the patients, as can be found in the literature [44,45,46,47,48,49,50], and is also consistent with the results obtained in other studies in which PRGF was used in patients with DED [23,24].

In the case of the SANDE questionnaires, there was a statistically significant improvement in the PRGF-treated group, with a large effect size, as can be seen in Figure 4. In order to obtain more details, we performed a subdivision according to the OSDI at the baseline visit, and this improvement, as can be seen in Table A1 and Figure A1 and Figure A2, was evident in the most severe cases.

It is well known that in DED there is a loss of ocular surface homeostasis with alteration in the tear composition and an increase in osmolarity causing inflammation and cell damage. Exposure of ocular surface epithelial cells to elevated tear osmolarity activates stress-associated mitogen-activated protein kinases [51]. Tear hyperosmolarity stimulates a cascade of events on the ocular surface involving the generation of inflammatory cytokines including interleukin-1 (IL-1), tumor necrosis factor-α (TNF-α), and proteases such as MMP-9. These activate and recruit inflammatory cells to the ocular surface, which become an additional source of inflammatory mediators [4]. Thus, the pro-inflammatory cytokines IL-1, TNF-α, IL-6, IL-23, IFN-γ, and IL-17 are elevated in DED [52].

This continuous process is called the vicious circle of DED [6]. To stop this cycle and prevent disease progression, it is crucial to resolve the initial stages of inflammation [53]. Therefore, the temporary use of corticosteroids is indicated as a treatment for DED to reduce inflammation, as was demonstrated by Marsh and Pflugfelder in 1999 [54]. The problem with the use of corticosteroids is their side effects, such as cataract formation, increased intraocular pressure, or facilitation of infection [54].

Regarding inflammation, the most common suggestive clinical sign in the eye may be conjunctival redness [32,33,55]. Ocular redness can be measured by subjective scales such as the McMonnies scale [56], which was the first to be used, or the Efron scale [57], which is widely used in daily clinical practice. The limitation of subjective scales is that they have a wide intra-and inter-observer variability [58,59]. This variability seems to be controlled by the use of automated quantification. Thus, Wu et al. concluded that the Keratograph^®^ is a reliable tool for scoring ocular redness using the Oculus Index, as it showed a significantly high level of reproducibility compared to subjective image-based scales [34].

Our research showed a statistically significant improvement in ocular redness in the PRGF group after treatment, with a significantly larger effect size compared to the standard group, as shown in Figure 4. When the variation of hyperemia was analyzed as a function of the initial OSDI value, it was found that inter- and intra-group improvements were obtained in moderate (large effect size) and severe cases, as shown in Table A2 and Figure A3. These findings may justify the use of PRGF in moderate to severe cases of ocular symptomatology and inflammation associated with DED, which is consistent with studies proposing the use of PRGF to treat inflammation [5]. Recently, Barros et al. demonstrated in a confocal microscopy study the decreased presence of inflammatory cells in evaporative DED patients treated with PRGF [60]. The observed decrease in ocular redness in patients treated with PRGF could be justified by the anti-inflammatory properties of TFG-β present in PRGF [18,61]. It is remarkable that, even with small variations in the Oculus Index, the clinical appearance is significant, as can be seen in Figure 3.

The condition of DED causes discomfort to patients as observed in daily clinical practice and as reported in the results of the symptomatology questionnaires. Inadequate hydration of the ocular surface, either due to a lack of tear secretion or excessive evaporation, increases the frequency of blinking, as has been previously studied, and although there is disagreement about the mechanism for this to occur (ophthalmologists attribute it to local mechanisms and neurologists attribute it to the central nervous system), Tsubota et al. demonstrated that the blink interval was increased in patients with DED compared to healthy patients. They also showed that the duration of the blink was longer in patients with DED [62]. This proves that patients with DED close their eyes for longer periods of time, perhaps in an involuntary attempt to reduce discomfort. According to the results of our study, it was observed that the total number of blinks decreased in the PRGF group after treatment compared to the standard treatment patients, which objectively confirmed the improvement of symptoms reported by the questionnaires. This reduced need for blinking may be justified by the analgesic properties of PRGF due to the antinociceptive activity of plasma [63] and the anti-inflammatory effects mediated by the PRGF [64,65].

As in previous studies by Sánchez-Ávila et al. [27,66,67], the visual acuity improved in the PRGF group after treatment, possibly due to a regularization of the ocular surface, which could improve optical quality.

Regarding the analysis of the Schirmer’s test, it was observed that there was no variability between groups, although a decrease was found in the standard group. At this point, it should be considered that although the Schirmer’s test is the gold standard in tear volume quantification, there are studies suggesting that it is a test with high variability [68,69]. This variability was also reported by Alió et al. [23] when they studied FBUT in patients treated with PRGF, in which 50% of the patients improved their values by 2 s and the other 50% remained the same. In our case, there was an improvement in the PRGF group, but it was not superior to the standard group.

There were also no differences in corneal staining, but there was a trend toward an improvement in the ocular surface in patients treated with PRGF, which may have contributed to the improvement in the BCVA achieved in the PRGF-treated group.

Further studies should improve on the limitations of the present work, such as the fact that it is retrospective and not controlled or segmented by more subgroups, including the study of previous treatments as well as the analysis of corneal innervation by confocal microscopy. Another bias to consider is the sample size. In addition, the PRGF group received this treatment by the ophthalmologist according to two criteria, one of which was that previous treatments had failed or that they had at least a moderate OSDI score. Although it was not statistically significant, the OSDI score was higher in the first visit in the PRGF group, which could be explained by the fact that an ophthalmologist in a clinical practice might prescribe this treatment in cases with higher subjective complaints. Even when our results showed no differences in baseline measures between patients in the PRGF group, some of the patients in this group had received other previous treatments, which may be a limitation of this study. Another limitation is that the sample consisted mainly of women, which could be explained by the higher prevalence of DED in women compared to men [70,71], although this is consistent with other similar studies [23].

## 5. Conclusions

This study fulfills one of the requirements of DEWS II: to investigate how the new platelet preparations affect the different variables in DED. According to the results obtained, the addition of PRGF in the treatment of dry eye was found to improve the symptomatology and reduce the amount of blinks and ocular inflammation, especially in cases of moderate and severe symptomatology, and no adverse effects were found. In addition, this is the first time that inflammation has been analyzed according to OSDI categories.

## Figures and Tables

**Table 1:** Summary of demographic data and inclusion criteria for analysis of the standard and PRGF groups and comparison of both groups at the baseline visit.

	Standard	PRGF	
	Mean ± SEM	Mean ± SEM	*p*-Value
*n* (right eye/left eye)	43 (22/21)	57 (29/28)	0.894
Gender (male/female)	9/13	3/27	0.009 *
Age	60.73 ± 3.11	54.47 ± 3.10	0.169
OSDI	36.36 ± 3.66	47.70 ± 4.38	0.151
FBUT	4.05 ± 0.48	5.44 ± 0.55	0.106
Local corneal staining	1.23 ± 0.07	1.24 ± 0.09	0.449

SEM (Standard Error of Mean), OSDI (Ocular Surface Disease Index), FBUT (Fluorescein Break-Up Time). * Statistically significant difference (*p* < 0.05).

**Table 2:** Previous treatment that the patients had received.

	AT	TC	PHP	PP	C
Standard	100% (*n* = 22)	0% (*n* = 0)	18.22% (*n* = 4)	0% (*n* = 0)	0% (*n* = 0)
PRGF	100% (*n* = 30)	20% (*n* = 6)	20% (*n* = 6)	3.3% (*n* = 1)	3.3% (*n* = 1)

AT (artificial tears), TC (topical corticosteroids), PHP (palpebral hygiene protocol), PP (punctal plugs), C (cyclosporine).

**Table 3:** General results.

	Standard Group				PRGF Group			
	Baseline	Follow-Up			Baseline	Follow-Up		
	Mean (±SEM)	Mean (±SEM)	*p*-Value	Effect Size ^+^	Mean (±SEM)	Mean (±SEM)	*p*-Value	Effect Size ^+^
OSDI Score	36.36 ± 3.66	21.81 ± 2.78	<0.0001 *	0.94	47.70 ± 4.38	34.32 ± 3.90	0.0003 *	0.59
SANDE Frequency	68.64 ± 4.95	45.45 ± 5.38	0.0006 *	0.93	78.97 ± 4.62	35.00 ± 3.90	<0.0001 *	2.05
SANDE Severity	58.18 ± 6.17	39.77 ± 5.49	0.0011 *	0.64	68.62 ± 4.25	29.66 ± 2.95	<0.0001 *	2.11
BCVA (LogMAR)	0.02 ± 0.008	0.01 ± 0.008	0.1316	0.20	0.08 ± 0.03	0.05 ± 0.02	0.0165 *	0.10
Total blinks (*n*)	7.61 ± 0.73	8.95 ± 0.69	0.1290	0.29	10.35 ± 0.81	8.46 ± 0.70	0.229 *	0.33
Ocular Redness (Index)	1.26 ± 0.07	1.27 ± 0.08	0.9161	0.02	1.35 ± 0.08	1.05 ± 0.06	<0.0001 *	0.57
Corneal Staining (Score)	1.23 ± 0.07	1.28 ± 0.07	0.7539	0.11	1.24 ± 0.09	1.18 ± 0.07	0.2876	0.13
FBUT (s)	4.05 ± 0.48	4.93 ± 0.42	0.0611	0.30	5.44 ± 0.55	6.81 ± 0.57	0.0006 *	0.34
Schirmer (mm)	6.74 ± 0.84	5.21 ± 0.68	0.0298 *	0.30	5.88 ± 0.64	5.93 ± 0.70	0.9402	0.01

SEM (Standard Error of Mean), OSDI (Ocular Surface Disease Index), SANDE (Symptom Assessment in Dry Eye), BCVA (Best Corrected Visual Acuity), FBUT (Fluorescein Break-Up Time). * Statistically significant difference (*p* < 0.05). ^+^ Hedges’ g.

## Data Availability

The data used to support this study’s findings are available by contacting the corresponding author upon reasonable request.

## Appendix A

Frequency and severity of symptoms according to the severity of the OSDI questionnaire at baseline: see Figure A1 and Figure A2. An improvement in the frequency and severity of symptoms was obtained in both groups, with a difference between groups (*p* < 0.05) in the case of initial severe OSDI. A large effect size was observed in PRGF-treated cases with moderate and severe baseline OSDI, see Table A1.

**Table A1:** Difference between baseline and follow-up visits of the standard and PRGF groups, according to the initial OSDI value.

	Standard Group	PRGF Group		
	Mean ± SEM	Mean ± SEM	*p*-Value	g +
SANDE frequency				
OSDI mild	−45.00 ± 10.25	−30.00 ± 20.25	0.8175	0.38
OSDI moderate	−6.00 ± 15.68	−50.00 ± 11.83	0.0556	1.28
OSDI severe	−21.25 ± 5.71	−46.05 ± 4.89	0.0030 *	1.16
SANDE severity				
OSDI mild	−38.00 ± 8.00	−21.00 ± 11.45	0.2381	0.67
OSDI moderate	7.00 ± 21.54	−46.00 ± 11.66	0.0624	0.91
OSDI severe	−20.83 ± 5.18	−41.84 ± 5.80	0.0183 *	0.90

SEM (Standard Error of Mean), OSDI (Ocular Surface Disease Index), SANDE (Symptom Assessment in Dry Eye). * Statistically significant difference (*p* < 0.05). + Hedges’ g.

## Appendix B

Ocular redness according to the severity of the OSDI questionnaire at baseline, see Figure A3. An improvement in ocular redness was obtained in the PRGF group, with a difference between groups (*p* < 0.05) in the case of initial moderate and severe OSDI. A large effect size was observed in PRGF-treated cases with moderate baseline OSDI, see Table A2.

**Table A2:** Difference between baseline and follow-up visits of ocular redness of standard and PRGF groups, according to baseline OSDI value.

	Standard Group	PRGF Group		
Ocular Redness	Mean ± SEM	Mean ± SEM	*p*-Value	G +
OSDI mild	−0.08 ± 0.11	−0.26 ± 0.17	0.3851	0.38
OSDI moderate	−0.06 ± 0.12	−0.51 ± 0.16	0.0434 *	0.95
OSDI severe	0.08 ± 0.10	−0.25 ± 0.08	0.0177 *	0.34

SEM (Standard Error of Mean), OSDI (Ocular Surface Disease Index), SANDE (Symptom Assessment in Dry Eye). * Statistically significant difference (*p* < 0.05). + Hedges’ g.
